# Expression of a mitochondrial gene *orfH79* from CMS-Honglian rice inhibits *Escherichia coli* growth via deficient oxygen consumption

**DOI:** 10.1186/s40064-016-2822-0

**Published:** 2016-07-19

**Authors:** Xia Ding, Qiusheng Chen, Canming Bao, Aihua Ai, Ying Zhou, Shaobo Li, Hongwei Xie, Youlin Zhu, Yaohui Cai, Xiaojue Peng

**Affiliations:** Key Laboratory of Molecular Biology and Gene Engineering of Jiangxi Province, College of Life Science, Nanchang University, Nanchang, 330031 People’s Republic of China; Jiangxi Super-Rice Research and Development Center, Nanchang, 330200 People’s Republic of China

**Keywords:** Cytoplasmic male sterility, *orfH79*, *E. coli* growth, Oxygen respiration

## Abstract

Cytoplasmic male sterility (CMS) has often been associated with abnormal mitochondrial open frames (ORF), *orfH79* is a mitochondrial chimeric gene responsible for the CMS trait in Honglian (HL) rice. In this study, the weakly produced ORFH79 protein significantly inhibited the growth of *E. coli* in an oxygen culture, however, the growth of the transformants producing ORFH79 was indistinguishable from the control under anaerobic incubation conditions. In addition, a lower respiration rate, wrinkled bacterial surfaces, and decreased pyruvate kinase and α-ketoglutarate dehydrogenase activities were observed in the ORFH79 produced *E. coli*. These results indicate that ORFH79 impairs the oxygen respiration of *E. coli*, which may inhibit *E. coli* growth.

## Background

Cytoplasm male sterility (CMS) is a widespread phenomenon in the plant kingdom. It is maternally inherited and characterized by a failure to produce functional pollen (Gray 1999; Young et al. 1987). In most cases, the failure of pollen development in CMS background is associated with chimeric mitochondrial open reading frames (ORFs) arising from unusual recombination events (Hanson and Bentolila 2004). In previous studies, many CMS-associated genes such as T-maize *urf13*, sunflower *orf522*, Brassica Ogura radish *orf138* and BT-rice *orf79* have been shown to encode peptides that are lethal to *E. coli* (Dewey et al. 1988; Duroc et al. 2005; Nakai et al. 1995; Wang et al. 2006). However, the mechanism by which this occurs remains relatively unknown. In addition, whether such a phenomenon is related to the mechanism of CMS has not yet been reported.

Honglian cytoplasmic male sterility (CMS-HL) rice (*Oryza sativa*) is one of the three typical CMS systems of rice. A mitochondrial chimeric gene named *orfH79*, located downstream of *atp6*, has been proved to result in the pollen abortion of CMS-HL rice (Peng et al. 2004; Yi et al. 2002). Moreover, transgenic *OrfH79* rice showed the accumulation of high levels of ROS, significantly decreased adenylate content, and ATP/ADP ratios, and reduced mitochondrial membrane potential, which mimicked CMS-HL rice (Peng et al. 2004). These physiological features suggest that *orfH79* expression impairs mitochondrial function. However, the mechanism by which the aberrant ORFH79 protein affects mitochondrial activity requires further investigation.

Due to the pollen-specific phenotype and because it is not technically feasible to handle plant mitochondria, the CMS mechanism is difficult to study. Usually, the expression pattern of a mitochondrial gene is similar to that *of E. coli* (Gray 1999). In this paper, we introduced the *orfH79* gene into *E. coli*, and we found that the weakly produced ORFH79 protein significantly inhibits the growth of *E. coli* in an oxygen culture, however, the growth of the transformants producing ORFH79 was indistinguishable from the control under anaerobic incubation conditions. In addition, a lower respiration rate, wrinkled bacterial surfaces, and decreased pyruvate kinase and α-ketoglutarate dehydrogenase activities were observed in the ORFH79 produced *E. coli*. These results indicate that ORFH79 impairs the oxygen respiration of *E. coli*, which may inhibit *E. coli* growth.

## Methods

### Strains and media

The strains used in this study, *E. coli* DH5α and BL21 (DE3) were stored in Key Laboratory of Molecular and Gene Engineering in Nanchang City. The strain was routinely grown in LB medium (5 g yeast extracts/l, 10 g peptone/l, and 10 g NaCl/l) at 37 °C with oxygen incubation. For anaerobic incubation, LB medium was prepared per 600-ml anaerobic bottle and sterilized under a strictly anaerobic H_2_ and CO_2_ atmosphere (80:20).

### Plasmid construction

The DNA fragment encoding *orfH79* was amplified from the CMS-HL rice by PCR using a primer set (forward, 5′ GCCGGATCCATGACAAATCTG CTCCGATGGCTC-3′; reverse, 5′ GCCCTCGAGTTACTTAGGAAAGACTACA CG-3′). The orfH79 gene was ligated to the *pET*-*28a* vector (Invitrogen) digested with NcoI and XholI to construct the plasmid *pET*-*28a*-*orfH79*. The *BL21* (DE3) strains were transformed with plasmids *pET*-*28a*-*orfH79* and *pET*-*28a* empty vector. As a control in some experiments, we also used *pET*-*28a* vector expressed a disulfide isomerase-like protein (PDI) gene, *MTH1745*, which contained a hygrophobic transmembrane structure from 10 to 30 amino acids in this protein (Ding et al. 2008).

### Preparation of cell fraction

Crude membrane versus cytoplasmic cell fraction were prepared described as Arockiasamy and Krishnaswamy (2000) with some modifications. 100 ml of bacterial cell culture were harvested and centrifuged at 10,000×*g* for 5 min at 4 °C. The precipitation was washed twice with phosphate buffered saline (PBS), and resuspended in the same buffer containing 0.1 M PMSF and 0.1 M PI (both Roche Diagnostics). The suspension was ultrasonicated on ice for 15 min (5 s on with 5 s intervals). Cell debris and insoluble proteins were recovered by centrifugation for 1 h at 10,000×*g*, and the supernatant was centrifuged at 30,000×*g* for 2 h at 4 °C. The supernatant was retained for further analysis and the pellet containing the crude membrane was resuspended in 200 µl dilution buffer (66 mM Tris–Cl, pH 6.8, 2 % v/v 2-mercaptoethanol, 2 % SDS).

### Antibodies and western blot analysis

Equal amounts of protein from the membrane and cytoplasm were separated by 18 % SDS-PAGE gel at 4 °C, and then transferred onto an Immobilon-PSQ transfer membrane (PVDF type; Millipore) at 80 V for 40 min. The membrane was incubated in 5 % w/v non-fat milk, 0.05 % v/v Tween-20, in PBS for 1 h, washed for 10 min three times in PBST (PBS, 0.05 %v/v Tween-20), and incubated in a 1:5000 dilution of mouse antiserum anti-ORFH79 overnight at 4 °C. After four washes with PBST, the membrane was incubated with rabbit anti-mouse IgG conjugated with alkaline phosphatase (AP) in PBST solution for 2 h. After four 10-min washes in PBST, the signal was visualized by chemiluminescent detection (Pierce, Rockford Rockford, IL) according to the manufacturer’s protocol. Antibody against ORFH79 was a gift from professor Shaoqing Li, College of Life Science, Wuhan University.

### *Escherichia coli* growth curve assay

The growth curve assay was performed to ascertain the effect of expressing *OrfH79* in *E. coli* cells were transformed with either *pET*-*28a*-*orfH79* or *pET*-*28a*. The transformants were incubated in LB medium at 37 °C overnight. For oxygen incubation, 30 ml LB medium supplemented with 30 µl preculture was incubated at 37 °C, with shaking. At an OD_600_ of 0.6, the culture was separated into two equal subcultures. IPTG was added to one of the cultures, at a final concentration of 1 mM. The cultures were grown at 37 °C with shaking (250 rpm), and the cell density (OD_600_) of these culture were monitored by withdrawing aliquots at various times. For anaerobic incubation, 600 µl overnight preculture was added to LB medium prepared per 600-ml anaerobic bottle and autoclave under a strictly anaerobic H_2_ and CO_2_ atmosphere (80:20), and incubated at 37 °C, with shaking. At an OD_600_ of 0.3, the culture was separated into two equal subcultures. IPTG was added to one culture, at a final concentration of 1 mM. The cultures were grown at 37 °C with shaking (250 rpm), and the cell density (OD_600_) of these culture were monitored by withdrawing aliquots at various times.

### Respiration measurement

BL21(DE3) pLYsS cells containing *pET*-*28a*-*orfH79* and *pET*-*28a* were cultured in 5 ml LB medium supplemented with antibiotics at 37 °C. Each preculture was diluted 1:1000 in fresh LB medium containing antibiotics. The resulting culture was incubated at 37 °C until the cells reached the early exponential growth phase. Each culture was then divided into two subcultures. One subculture was induced with 1 mM IPTG whereas the other was used as a control. Both subcultures were incubated at 37 °C, with shaking, for an additional 2 h. Cellular respiration was measured at HPES-KPR buffer (50 mM HPES, pH7.4, 100 mM NaCL, 5 mM KCL, 1 mM MgCL_2_, 1 mM NaH_2_PO_4_, 1 mM d-glucose and 1 mM CaCl_2_). Respiration was measured using the temperature-controlled chamber of a chlorolab 2 electrode (Hansatech, UK) containing 2 ml of buffer. The respiration rate was measured by the detection of oxygen consumption at 37 °C.

### Membrane observation of *E. coli* by scanning electron microscopeye

The bacterial cells were fixed with 2.5 % glutaradehyde overnight at 4 °C. The fixed cells were washed three times with phosphate buffer (0.1 M, pH 7.0), 15 min each time. The bacterial cells were dehydrated in ascending concentration of ethanol (10, 20, 30, 40, 50, 60, 70, 80, 90, 95, 100 and 100 %) at 15 min exposure for each concentration. The bacterial cells were further dehydrated in different ratios of ethanol: acetone (3:1, 1;1 and 1:3) for 20 min for each mixture and then washed with pure acetone four times each for 20 min. These bacterial cells were subjected to critical point drying using liquid CO_2_ and the cells mounted on a stub. These cells were coated with gold and examined by using scanning electron microscope.

### Detection of pyruvate kinase and α-ketoglutarate dehydrogenase activity

BL21(DE3) pLYsS cells, harboring the expression vectors *pET*-*28a*-*orfH79* and *pET*-*28a*, were cultured in LB medium until the optical density reached 0.6–0.8. Each culture was then divided into two subcultures. One subculture was induced with 1 mM IPTG whereas the other was used as a control. Cells were harvested at 6 h and 12 h, after induction with IPTG. Cells were disrupted by sonication and centrifuged to remove cell debris. The supernatants were used to determine the protein quantity and for use in enzymatic assays. Pyruvate kinase activity was measured using the ultraviolet chromatometry method following the protocol of the pyruvate kinase activity assay kit (Nanjing Jiancheng, China). The α-ketoglutarate dehydrogenase activity determination was performed described by Flora (Pettit et al. 1973).

### Statistical analysis

All experimental data are the mean of at least three independent replicates, and comparisions between transformants were performed using one way ANOVA with Duncan’s multiple range test. All the statistical analyses were performed using SPSS software.

## Results

### ORFH79 protein is primarily localized in the membrane of *E. coli*

**Fig. 1:**
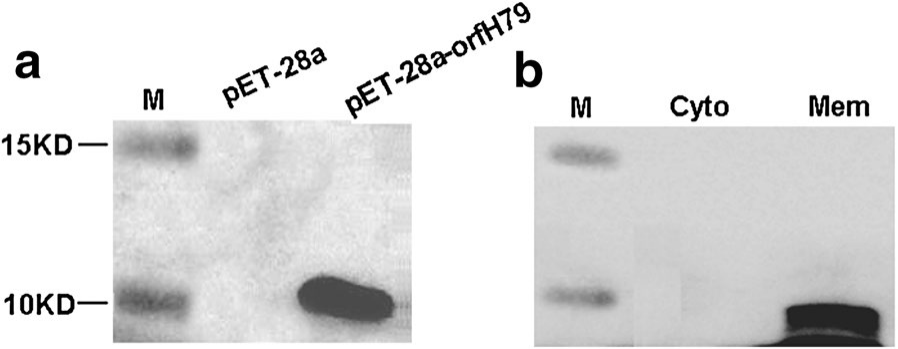
Western blot analysis of ORFH79 in whole *E. coli* extracts and fractions. **a** Detection of ORFH79 in whole *E. coli* extracts. Total protein was extracts from *pET*-*28a* or *pET*-*28a*-*orfH79* transformants induced with 1 mM IPTG, and subjected to PAGE in a 15 % acylamide gel. Western blot was preformed to detect the production of ORFH79 protein. Signal were obtained in the protein fraction from *pET*-*28a*-*orfH79* transformants. No signal appeared in the protein fraction from *pET*-*28a* transformants. **b** Intracellular localization of ORFH79 in *E. coli*. Crude membrane versus cytoplasmic cell fraction were prepared from *pET*-*28a*-*orfH79* transformants by series of centrifugation and subjected to PAGE in a 15 % acylamide gel. Western blot was preformed to detect Intracellular localization of ORFH79 in *E. coli*. Signal were obtained in the crude membrane fraction. *M* marker; *Cyto* Cytoplasmic fraction of *pET*-*28a*-*orfH79* transformants; *Mem* Crude membrane fraction of pET-28*a*-*orfH79* transformants

We extracted the total protein from *E. coli* transformants after induction, and then preformed SDS-PAGE and western blot analysis. The ORFH79 protein was not observed on Coomassie-blue gels (data not shown), whereas the production of ORFH79 was confirmed by western blot assay (Fig. 1a). A band of approximately 9KD was detected in the total protein samples from *pET*-*28a*-*orfH79* transformed *E. coli*, and no signal was detected in *E. coli* transformed with the empty plasmid (*pET*-*28a*). In addition, the subcellular distribution of ORFH79 was also determined. As shown in Fig. 1b, ORFH79 is primarily distributed in the crude membrane protein fractions (Fig. 1b). These results indicate that the ORFH79 protein is mainly attached to the *E. coli* membrane.

### Expression of orfH79 inhibits the growth of *E. coli* in oxygen culture and has no negative effects on the growth of *E. coli* under anaerobic conditions

**Fig. 2:**
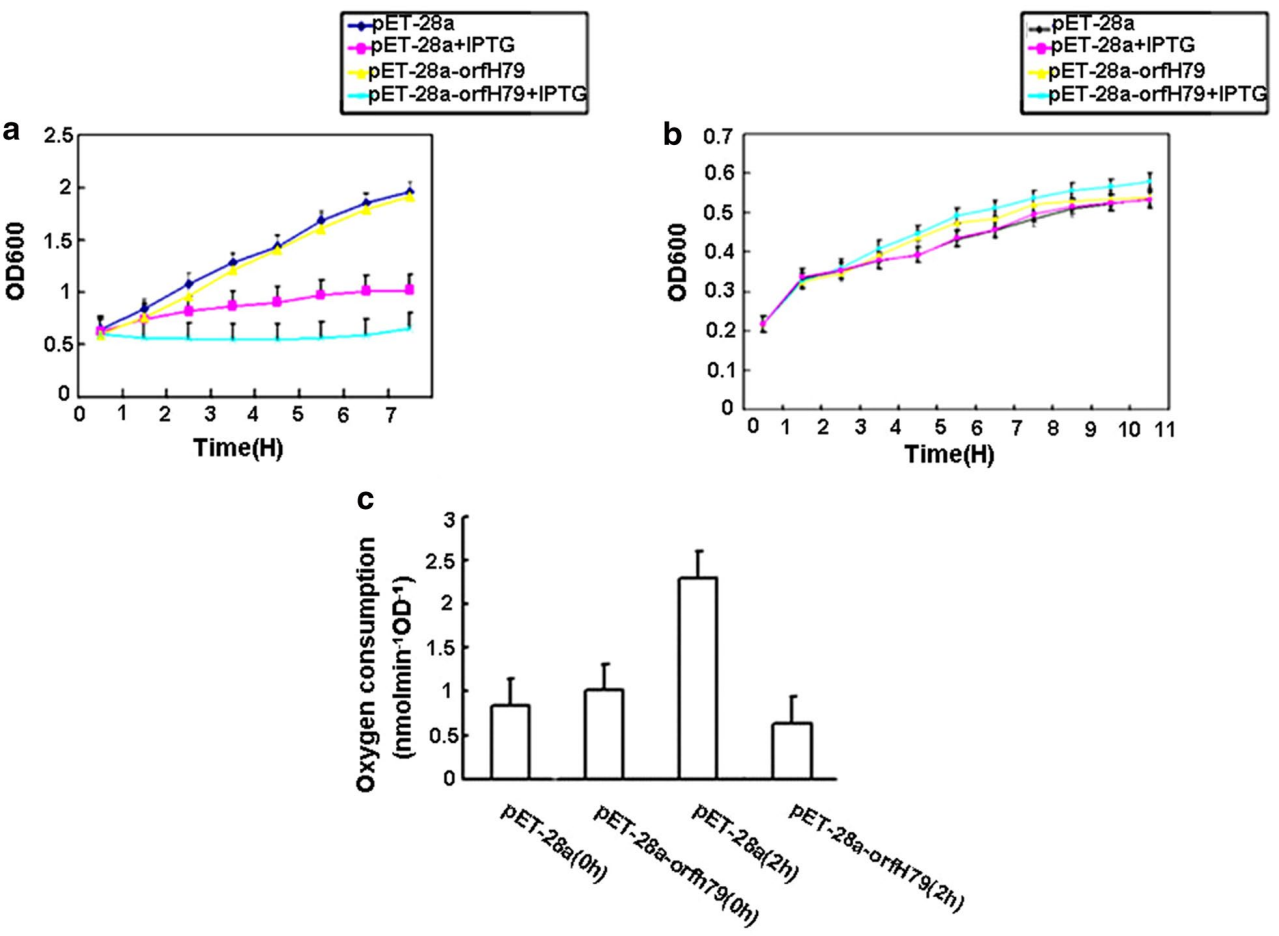
Growth curves and respiration measurements of transformants. **a** Growth curve of transformants in oxygen culture. **b** Growth curve of transformants with anaerobic induction. Cell density was determined at various time points at OD_600_. Cells were cultured at 30 °C. Data were presented as the mean ± SE of triplicate. **c** Oxygen consumption detection of transformants. *E. coli* cells was measured at the time of induction and 2 h later in 2 ml at 37 °C. The results are the mean of three measurements

During the induction of *orfH79* expression in oxygen culture, we found that the growth rate of the induced *pET*-*28a*-*orfH79* transformants was significantly inhibited compared to the control, and we then test the cell growth. As shown in Fig. 2a, transformants of *pET*-*28a*-*orfH79* and *pET*-*28a* exhibited similar growth rates in liquid medium without IPTG, whereas in the IPTG induced culture, ORFH79 production significantly decreased the growth rate of *E. coli* compared with the empty plasmid transformants. Additionally, no obvious exponential phase appeared in the growth curve of *pET*-*28a*-*orfH79* transformants in an oxygen culture. In this study, we found that the transformants producing ORFH79 protein have slow growth similar to fermentation growth, therefore, we monitored the anaerobic growth rate of these transformants. As shown in Fig. 2b, under anaerobic incubation conditions, growth curve of the induced *pET*-*28a*-*orfH79* transformants was similar to the control. These findings suggest that ORFH79 mediates inhibition of *E. coli* growth and is associated with the inhibition of oxidative phosphorylation.

### The respiration rate is reduced in orfH79 expressing transformants

Because ORFH79 is localized in the membrane of *E. coli,* and because *orfH79* expression inhibits *E. coli* growth in an oxygen culture, whereas it has no negative effects on the growth of *E. coli* under anaerobic incubation conditions, we focused on the respiration rate of the transformants. We used an oxygen electrode to measure the oxygen consumption of bacteria that did and did not produce ORFH79. Oxygen consumption was measured at induction and 2 h later. As shown Fig. 2c, the respiration rates of the *orfH79* expressing transformants were significantly decreased by about 65.2 % compared with the control, suggesting that oxygen consumption was affected by ORFH79 in *E. coli* cells.

### Wrinkled bacterial surfaces appear in ORFH79 producing *E. coli*

**Fig. 3:**
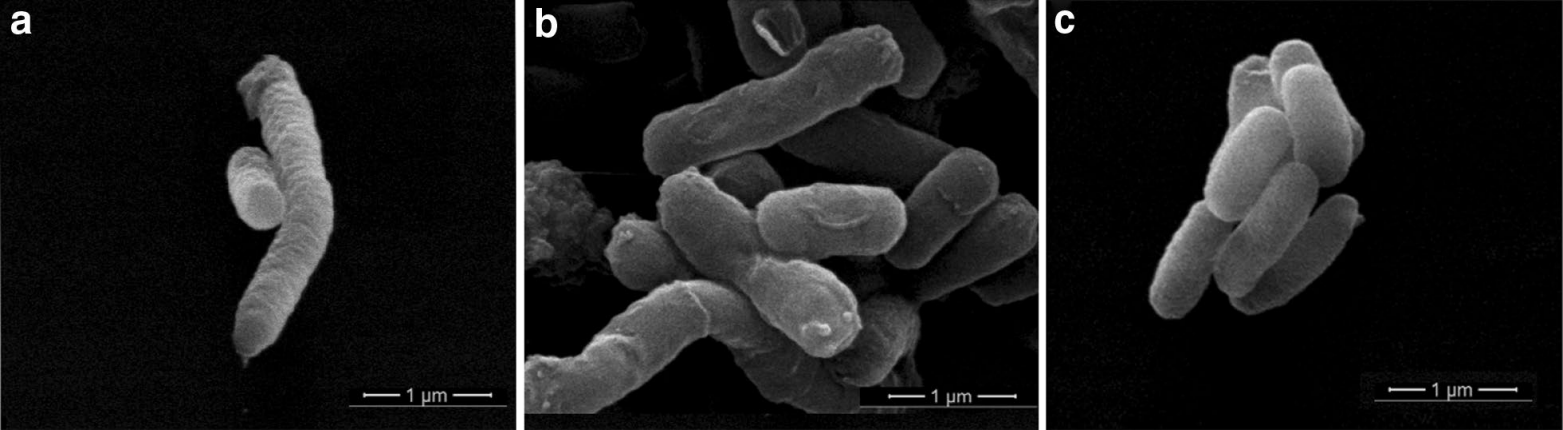
Scanning electron microscope images of transformants. **a** Images of ORFH79 protein produced *E. coli* cells. **b** Images of empty plasmid transformed *E. coli* cells. *Bar* 1 µm. **c** Images of MTH1745 protein produced *E. coli* cells

Oxygen consumption in *E. coli* cells occur via respiration chain, which is distributed in the bacterial plasma membrane. Although ORFH79 appear to weaken oxidative respiration, we want to know whether the membrane structure of the bacterial is normal, then the SEM was used to evaluate the ultrastructural changes of the cell membranes from bacteria that did and did not produce ORFH79. As shown in Fig. 3, the control cells had well-defined and smooth membranes, whereas ORFH79 production resulted in a wrinkled membrane surface. The winkled membrane is consistent with the dysfunction of respiration in ORFH79 producing *E. coli* cells, proving direct evidence that ORFH79 impairs the oxygen respiration of *E. coli*.

### ORFH79 production decreased pyruvate kinase and α-ketoglutarate dehydrogenase activity

**Fig. 4:**
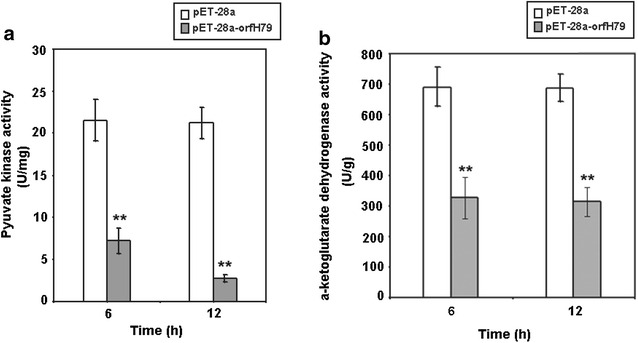
Pyruvate kinase and α-ketoglutarate dehydrogenase activity assay in transformants. **a** Pyruvate kinase activity assay in transformants. **b** The α-ketoglutarate dehydrogenase activity assay in transformants. Data were shown as the mean ± SE of triplicate. *Double asterisk* the difference between *pET*-*28a* and *pET*-*28*-*orfH79* transformants is significant (p < 0.01)

Oxidative phosphorylation involves a series of respiratory related enzymes. Pyruvate kinase and α-ketoglutarate dehydrogenase are two of important enzymes preserve the efficiency of oxygen respiration in the cell. Therefore, we monitored the activity of pyruvate kinase and α-ketoglutarate dehydrogenase in these transformants at 6 and 12 h of induction, respectively. The data show that the pyruvate kinase activity of ORFH79 producing transformants was significantly lower than the empty plasmid transformants at both 6 h and 12 h of induction later, and was twofold and ninefold lower than that of control respectively. The α-ketoglutarate dehydrogenase activity in ORFH79 producing transformants was significantly decreased by 52.8–54.4 % compared with the control after 6 and 12 h of induction. These results indicate that the production of ORFH79 caused decrease respiration related enzyme activity in *E. coli* (Fig. 4).

## Discussion

Recent reports about mechanism of CMS have provided insight into the mitochondrial respiratory chain complex. Luo et al. (2013) reported that WA352, a mitochondrial protein responsible for CMS of wild abortive rice, interacts with mitochondrial protein COX11. Furthermore, the ORFH79 protein, was founded to bind to mitochondrial complex III and decrease its enzyme interaction with P61 (Wang et al. 2013). These recent findings focus the attention regarding the mechanism of CMS mechanism on the interaction between CMS associated proteins and mitochondrial respiratory complex. However, it is not technically feasible to handle the pollen-specific phenotype, and the bioinformatics techniques for plant mitochondria must be improved to overcome the difficulties in studying plant pollen mitochondrial respiratory chain.

The respiratory complex is conserved between the *E. coli* membrane and mitochondria (Esterhazy et al. 2008; Grigorieff 1998; Guenebaut et al. 1998). In addition, the gene transcription and translation patterns in mitochondria are similar to *E. coli* (Gray 1999). Many reports indicate that the dysfunction of mitochondria in CMS plants is associated with the deficiency in oxidative phosphorylation,while direct evidence for this is lacking. Our experiments showed that the weakly produced ORFH79 protein significantly inhibits the growth of *E. coli* in oxygen culture, and was accompanied by a lower respiration rate, decreased pyruvate kinase and α-ketoglutarate dehydrogenase activity in *E. coli*, and wrinkled bacterial surfaces. Moreover, the growth of the ORFH79 producing transformants under anaerobic incubation conditions was not changed, providing direct evidence that ORFH79 impairs oxygen respiration. Additionally, it is notable that a typical exponential phase did not occur in the growth curve of ORFH79 producing *E. coli* in oxygen culture. Considering the deficient oxygen consumption with the increases demand for energy equivalents for rapid organism growth and division at the exponential stage, we infer that the expression of *orfH79* in *E. coli* impair the oxygen respiration such that the particularly high level energy demands for the *E. coli* cell growth and divided at the exponential stage can-not be met. Taken together, our find in the experiment provide more detailed information about the mechanism on CMS in Honglian rice using the *E. coli* model system.

## Conclusion

In summary, we found the weakly produced ORFH79 protein significantly inhibits the growth of *E. coli* in oxygen culture, and was accompanied by a lower respiration rate, decreased pyruvate kinase and α-ketoglutarate dehydrogenase activity in *E. coli*, and wrinkled bacterial surfaces. Moreover, the growth of the ORFH79 producing transformants under anaerobic incubation conditions was not changed. These results indicate that orfH79 protein impairs oxygen respiration, which may inhibit *E. coli* growth.

## References

[CR1] Arockiasamy A, Krishnaswamy S (2000). Purification of integral outer-membrane protein OmpC, a surface antigen from Salmonella typhi for structure-function studies: a method applicable to entero-bacterial major outer-membrane protein. Anal Biochem.

[CR2] Dewey RE, Siedow JN, Timothy DH, Levings CS (1988). A 13-kilodalton maize mitochondrial protein in *E. coli* confers sensitivity to Bipolaris maydis toxin. Science.

[CR3] Ding X, Lv ZM, Zhao Y, Min H, Yang WJ (2008). MTH1745, a protein disulfide isomerase-like protein from thermophilic archaea, *Methanothermobacter thermoautotrophicum* involving in stress response. Cell Stress Chaperones.

[CR4] Duroc Y, Gaillard C, Hiard S, Defrance MC, Pelletier G, Budar F (2005). Biochemical and functional characterization of ORF138, a mitochondrial protein responsible for Ogura cytoplasmic male sterility in Brassiceae. Biochimie.

[CR5] Esterhazy D, King MS, Yakovlev G, Hirst J (2008). Production of reactive oxygen species by complex I (NADH: ubiquinone oxidoreductase) from *Escherichia coli* and comparison to the enzyme from mitochondria. Biochemistry.

[CR6] Gray MW (1999). Evolution of organellar genomes. Curr Opin Genet Dev.

[CR7] Grigorieff N (1998). Three-dimensional structure of bovine NADH: ubiquinone oxidoreductase (complex I) at 22 A in ice. J Mol Biol.

[CR8] Guenebaut V, Schlitt A, Weiss H, Leonard K, Friedrich T (1998). Consistent structure between bacterial and mitochondrial NADH: ubiquinone oxidoreductase (complex I). J Mol Biol.

[CR9] Hanson MR, Bentolila S (2004). Interactions of mitochondrial and nuclear genes that affect male gametophyte development. Plant Cell.

[CR10] Luo D, Xu H, Liu Z, Guo J, Li H, Chen L, Fang C, Zhang Q, Bai M, Yao N, Wu H, Ji C, Zheng H, Chen Y, Ye S, Li X, Zhao X, Li R, Liu YG (2013) A detrimental mitochondrial-nuclear interaction causes cytoplasmic male sterility in rice. Nat Genet 45:573–577 10.1038/ng.2570 23502780

[CR11] Nakai S, Noda ND, Kondo M, Terachi T (1995). High-level expression of a mitochondrial orf522 gene from the male-sterile sunflower is lethal to *E. coli*. Breed Sci.

[CR12] Peng X, Wang K, Hu C, Zhu Y, Wang T, Yang J, Tong J, Li S (2004). The mitochondrial gene orfH79 plays a critical role in impairing both male gametophyte development and root growth in CMS-Honglian rice. BMC Plant Biol.

[CR13] Pettit FH, Hamilton L, Munk P, Namihira G, Eley MH, Willms CR, Reed LJ (1973). Alpha-keto acid dehydrogenase complexes. XIX. Subunit structure of the *Escherichia coli* alpha-ketoglutarate dehydrogenase complex. J Biol Chem.

[CR14] Wang Z, Zou Y, Li X, Zhang Q, Chen L, Wu H, Su D, Chen Y, Guo J, Luo D, Long Y, Zhong Y, Liu YG (2006). Cytoplasmic male sterility of rice with boro II cytoplasm is caused by a cytotoxic peptide and is restored by two related PPR motif genes via distinct modes of mRNA silencing. Plant cell.

[CR15] Wang K, Gao F, Ji Y, Liu Y, Dan Z, Yang P, Zhu Y, Li S (2013) ORFH79 impairs mitochondrial function via interaction with a subunit of electron transport chain complex III in Honglian cytoplasmic male sterile rice. New Phytol 198:408–418 10.1111/nph.12180 23437825

[CR16] Yi P, Wang L, Sun Q, Zhu Y (2002). Discovery of mitochondrial Chimeric gene associated with male sterility of HL-rice. Chin Sci Bull.

[CR17] Young JD, Cohn ZA, Gilula NB (1987). Functional assembly of gap junction conductance in lipid bilayers: demonstration that the major 27 kd protein forms the junctional channel. Cell.

